# 

**Published:** 1893-03

**Authors:** 


### Communicated.

WORLD'S COLUMBIAN COMMISSION—EXECUTIVE COMMITTEE ON AWARDS.—Upon January 14 last the rules and regulations to govern the matter of awards at the World's Exposition were adopted. These rules provide among other things that each exhibit shall be examined by an individual judge, who must be so far as possible a competent expert and must formulate his opinion of an exhibit in writing and sign his name thereto, and that this report must receive the confirmation of the Department Committee of which he is a member. There are thirteen of these Department Committees, one of which is assigned to each of the thirteen great Departments of the Exposition, and these thirteen committees compose the Board of Judges. There will be foreign representatives upon each of these thirteen committees, and also one or more women judges upon all committees authorized to award prizes for exhibits which may be produced in whole or in part by female labor.

It is the desire of the World's Columbian Commission to compensate judges, but this will depend upon the action of Congress, to which application has been made for an appropriation sufficient to defray all expenses connected with this subject of Awards. Should the appropriation be made, judges from the United States will receive $600 each, and those from foreign countries $1,000 each. Their work should begin June 1 and last about two months.

The management of this whole matter is in the hands of this Executive Committee on Awards, which is receiving applications for appointments as judges at their temporary offices and addresses as above. After March 15 the office will be permanently established in the Administration Building of the World's Columbian Commission, at Jackson Park, Chicage. As the judges are to be so far as possible competent experts, and the duties of the position require efficiency and ability of a very high order, and it is the desire of the Executive Committee to appoint only those absolutely qualified for the place and, whenever possible, of national and international reputation, this Executive Committee considers it its duty to call the attention of the technical journals of the country to the facts in order that it may receive the assistance and co-operation of those who by their position and connections are best able to assist the committee. It is not possible to make this announcement in the way of an advertisement, and the information is sent you in the belief that it is of sufficient interest to your readers to obtain some form of mention in your journal, and that you will suggest the names of such experts as you know to be of the capacity required.

JOHN BOYD THACHER,
*Chairman Executive Committee on Awards.*

### THE FIRST PAN-AMERICAN MEDICAL CONGRESS.

SECTION ANNOUNCEMENT OF THE FIRST PAN-AMERICAN MEDICAL CONGRESS, TO BE HELD AT WASHINGTON, D. C., SEPTEMBER 5, 6, 7 AND 8, A. D. 1893.

President: William Pepper, M. D., LL. D., 1811 Spruce Street, Philadelphia, Pa.; Secretary General: Charles A. L. Reed, M. D., 311 Elm St., Cincinnati, O.; Treasurer: A. M. Owen, M. D., 507 Upper Front St., Evansville, Ind.; Chairman of Executive Committee: Dr. Henry D. Holton, Brattleboro, Vt.

Committee of Arrangements: (Office, Arlington Hotel, Washington, D. C.) Sam'l. S. Adams, M. D., Chairman, 1632 K St., Washington, D. C.; J. R. Wellington, M. D., Secretary, 1406 Fifteenth St., Washington, D. C.

Cincinnati, O., January 31, 1893.

To the Dental Profession of the Western Continent:

The Pan-American Medical Congress to meet in Washington, D. C., September 5 to 8, 1893, being an assured success, the Dental Section promises to be well represented.

No other section of the Congress can claim a greater number of men of scientific attainment. In artistic and mechanical skill, in accurate and delicate manipulation where surgery is involved, in Bacteriology and Histology and rapid progress in its speciality, no other surpasses that of the Dental profession. Able papers of the following subjects will attest the above assertion. Cleft Palate, Hare Lip, Orthodontia, Dental Anatomy, Histology and Pathology; new growths of every character pertaining to the mouth and teeth. Diseases of the maxillary sinus and alveolar processes, Periostitis, Pulpitis and their results, Operative Dentistry, Bacteriology, Mechanical Dentistry, in addition to many other suitable topics.

This Congress, being an out-growth of the American Medical Association, the requirements for membership in this section are identical with that of the A. M. A., viz.; Any reputable practitioner holding the title of D. D. S. or M. D. S. can become a member the same as if he possessed the M. D.

To members of the profession in our sister countries we extend a hearty invitation to visit us and participate in the meeting, either by writing papers or by being present to hear or discuss them. This is especially desirable since the Congress belongs equally to all American countries. Many of you will, no doubt, visit the great World's Fair. This is also the year for the World's Columbian Dental Congress in Chicago This meeting, however, in no way interferes with the P. A. M. C., since the Columbian comes August 14 to 19 inclusive and the P. A. M. C., September 5 to 8 inclusive in Washington, thus offering two attractions in the way of Scientific Dentistry in addition to the Great Fair. Many of the officers of one Congress are officially connected with the other.

To the Columbian you are an invited guest, at the Pan-American you are participating in an institution as much your own as ours.

To the Dental profession in the United States we would suggest that in taking part in this, the first meeting of the P. A. M. C., we are the hosts, and our duties as such need not be rehearsed.

The excess of Dental practitioners in the United States over our sister countries will necessitate a careful selection of topics and papers in order to present the highest standard, the object being not numbers, but quality of material and ample time for discussion.

The social feature of the Congress will be no small part of the attractions.

Respectfully,

M. H. FLETCHER,

Executive President of the Section on Oral and Dental Surgery of the P. A. M. C.

### EXTRACTS FROM REGULATIONS AND BY-LAWS.

### MEMBERSHIP.

Members of the Congress shall consist of such members of the medical profession of the Western Hemisphere, including the West Indies and Hawaii, as shall comply with the special regulations regarding registration, or who shall render service to the Congress in the capacity of Foreign Officers. [*General Regulation 2.*]

### CONSTITUENT COUNTRIES.

The following shall be considered as the constituent countries of the Pan-American Congress.

Argentine Republic, Bolivia, Brazil, British North America, British West Indies, (including B. Honduras), Chile, Dominican Republic, Honduras (Sp.,) Mexico, Nicaragua, Paraguay; Peru, Salvador, Republic of Columbia, Republic of Costa Rica, Ecuador, Guatemala, Haiti, Kingdom of Hawaii, Spanish West Indies, United States, Uruguay, Venezuela, Danish, Dutch and French West Indies. [*General Regulation 7.*]

### SECTIONS.

The Sections of the Congress shall be as follows:

(1) General Medicine, (2) General Surgery, (3) Military Medicine and Surgery, (4) Obstetrics, (5) Gynæcology and Abdominal Surgery, (6) Therapeutics, (7) Anatomy, (8) Physiology, (9) Diseases of Children, (10) Pathology, (11) Opthalmology, (12) Laryngology and Rhinology, (13) Otology, (14) Dermatology and Syphilography, (15) General Hygiene and Demography, (16) Marine Hygiene and Quarantine, (17) Orthopædic Surgery, (18) Diseases of the Mind and Nervous System, (19) Oral and Dental Surgery, (20) Medical Pedagogics, (21) Medical Jurisprudence, (22) Railway Surgery. [*General Regulation 8.*]

### LANGUAGES.

The languages of the Congress shall be Spanish, French, Portuguese and English. [*General Regulation 9.*]

### REGISTRATION.

The Registration fee shall be $10.00 for each member residing in the United States, but no fee shall be charged to foreign members. Each registered member shall receive a card of membership and be furnished a set of the transactions. [*Special Regulation 2.*]

ABSTRACTS, PAPERS AND DISCUSSIONS.

Contributors are required to forward abstracts of their papers, not to exceed six hundred words each, to be in the hands of the Secretary-General not later than the tenth of July, 1893. These abstracts shall be translated into English, French, Spanish and Portuguese, and shall be published in advance of the meeting for the convenience of the Congress, and no paper shall be placed upon the program which has not been thus presented by abstract. Abstracts will be translated by the Literary Bureau of the Congress at the request of contributors, and should be forwarded through the Secretaries of Sections. Papers to be presented to Sections must not consume more than twenty minutes each in reading, and when of greater length must be read by abstract not exceeding twenty minutes in length. Papers read by abstract may be printed in full in the transactions, subject to approval by the Editorial Committee. Papers and discussions will be printed in the language in which they may be presented. All papers read in the Sections shall be surrendered to the Secretaries of the Sections; all addresses read in the General Session shall be surrendered to the Secretary-General as soon as read; and all discussions shall be at once reduced to writing by the participants. [*Special Regulation 3.*]

LITERARY BUREAU.

The Secretary-General may at his discretion organize a Literary Bureau, which shall consist of such number of linguists as he may determine, whose duty it shall be to do all necessary translating for the Congress, compensation for which service shall be determined by the Executive Committee. Certain members of the Literary Bureau may be designated by the Secretary-General as an Editorial Commttee. It shall be the duty of the Editorial Committee to determine the eligibility of all contributions before the same shall be published in the Transactions, and to supervise the publication of both the Book of Abstracts and the transactions. All work done by the Editorial Committee and by the Literary Bureau shall be subject to approval by the Secretary-General. [*By law V.*]

---

### Editorial, Etc.

THE AMERICAN MEDICAL CONGRESS.--Especial attention is invited to the section announcement of the first Pan-American Medical Congress to be held in Washington, D. C., next September. This Congress is an outgrowth of the American Medical Association, in which the Section of Oral and Dental Surgery was organized about twelve years ago. "Any reputable practitioner holding the title of D. D. S., or M. D. S., can become a member the same as if he possessed the M. D."

The papers discussed at the meetings of the Section of Oral and Dental Surgery of the American Medical Association, as is well known to readers of dental literature, have commanded the attention and respect of the medical profession. It is, therefore, fair to anticipate a profitable meeting in September, inasmuch as some of the officers of the Dental Section of the proposed Pan-American Congress are leaders of thought in the dental profession in this country, and have been active workers in and contributors to the Section of Oral and Dental Surgery of the American Medical Association in which they are recognized as men of unusual scientific ability.

